# Neutrophil to lymphocyte ratio predicts intracranial hemorrhage after endovascular thrombectomy in acute ischemic stroke

**DOI:** 10.1186/s12974-018-1359-2

**Published:** 2018-11-15

**Authors:** Slaven Pikija, Laszlo K. Sztriha, Monika Killer-Oberpfalzer, Friedrich Weymayr, Constantin Hecker, Christian Ramesmayer, Larissa Hauer, Johann Sellner

**Affiliations:** 1 Department of Neurology, Christian Doppler Medical Center, Paracelsus Medical University, Ignaz-Harrer-Straße 79, 5020 Salzburg, Austria; 2 Department of Neurology, King’s College Hospital, Denmark Hill, London, UK; 3 Research Institute for Neurointervention, Christian Doppler Medical Center, Paracelsus Medical University, Salzburg, Austria; 4 Division of Neuroradiology, Christian Doppler Medical Center, Paracelsus Medical University, Salzburg, Austria; 5 Department of Psychiatry, Psychotherapy and Psychosomatics, Christian Doppler Medical Center, Paracelsus Medical University, Salzburg, Austria; 6 Department of Neurology, Klinikum rechts der Isar, Technische Universität München, Munich, Germany

**Keywords:** Ischemic stroke, Inflammation, Intracranial hemorrhage, Thrombectomy, Outcome

## Abstract

**Background:**

The development of intracranial hemorrhage (ICH) in acute ischemic stroke is associated with a higher neutrophil to lymphocyte ratio (NLR) in peripheral blood. Here, we studied whether the predictive value of NLR at admission also translates into the occurrence of hemorrhagic complications and poor functional outcome after endovascular treatment (EVT).

**Methods:**

We performed a retrospective analysis of consecutive patients with anterior circulation ischemic stroke who underwent EVT at a tertiary care center from 2012 to 2016. Follow-up scans were examined for non-procedural ICH and scored according to the Heidelberg Bleeding Classification. Demographic, clinical, and laboratory data were correlated with the occurrence of non-procedural ICH.

**Results:**

We identified 187 patients with a median age of 74 years (interquartile range [IQR] 60–81) and a median baseline National Institutes of Health Stroke scale (NIHSS) score of 18 (IQR 13–22). A bridging therapy with recombinant tissue-plasminogen activator (rt-PA) was performed in 133 (71%). Of the 31 patients with non-procedural ICH (16.6%), 13 (41.9%) were symptomatic. Patients with ICH more commonly had a worse outcome at 3 months (*p* = 0.049), and were characterized by a lower body mass index, more frequent presence of tandem occlusions, higher NLR, larger intracranial thrombus, and prolonged rt-PA and groin puncture times. In a multivariate analysis, higher admission NLR was independently associated with ICH (OR 1.09 per unit increase, 95% CI (1.00–1.20, *p* = 0.040). The optimal cutoff value of NLR that best distinguished the development of ICH was 3.89.

**Conclusions:**

NLR is an independent predictor for the development of ICH after EVT. Further studies are needed to investigate the role of the immune system in hemorrhagic complications following EVT, and confirm the value of NLR as a potential biomarker.

## Introduction

Stroke resulting from large vessel occlusion is a devastating disease with a mortality rate of up to 80% [1, 2]. Endovascular thrombectomy (EVT) significantly improves outcomes with almost half of the patients achieving functional independence at 90 days [3]. Indeed, benefits of EVT using second-generation devices over medical therapy alone in five clinical trials of stroke caused by large vessel occlusion were remarkable, and the procedure has been quickly implemented in clinical practice [4]. However, there are peri- and post-interventional complications that need to be considered. Recent EVT trials have reported an occurrence rate of any intracerebral hemorrhage (ICH) of up to 46% [5, 6]. Symptomatic ICH (sICH), however, occurs less frequently, as seen at 4.4% in the meta-analysis of the HERMES collaboration [7]. There is emerging evidence to indicate that reperfusion injury and ICH are facilitated by post-stroke immune responses, and its impact on the neurovascular interface. Inflammatory mediators involved in this response include cytokines, chemokines, adhesion molecules, and several immune molecule effectors such as matrix metalloproteinases-9, inducible nitric oxide synthase, nitric oxide, and reactive oxygen species [8, 9]. Of note, neutrophil to lymphocyte ratio (NLR) is seen as a systemic marker of subclinical inflammation, and an increased ratio is of prognostic value in several disorders [10]. Importantly, in-hospital mortality and poor outcome at 90 days in ischemic stroke is associated with a higher NLR on admission [11–13]. A NLR above 5.9 also predicted death and 90 day outcome after endovascular therapy for large vessel ischemic stroke [14]. Moreover, patients with NLR ≥ 4.8 before thrombolysis had a 3.71-fold increased risk for sICH [15]. Here, we hypothesized that NLR predicts non-procedural ICH in patients who undergo EVT for acute stroke caused by large artery occlusion in the anterior circulation.

## Subjects and methods

We performed a retrospective review of all consecutive stroke patients admitted to Christian Doppler Medical Center (Salzburg, Austria) from 2012 to 2016. The study protocol was in accordance with the guidance of our hospital’s committee for the protection of human subjects (protocol UN 2553). According to Austrian regulations, an informed consent is not required for routinely collected clinical and radiological data as used in this study. A written approval for the retrospective study of patients with acute ischemic stroke was obtained from the local ethics committee (415-EP/73/750-2017).

The inclusion criteria were as follows: ≥ 18 years of age, ICA and/or MCA occlusion confirmed by CT-angiography (CTA) or MR-angiography within 6 h from symptom onset, and EVT was performed with contemporary thrombectomy techniques. Imaging and EVT were conducted according to an in-house protocol regularly updated with published high-quality evidence, as reported previously [11]. A follow-up CT was performed routinely within 24 h from EVT, and thereafter on an individual basis in the case of any clinical deterioration to determine presence of sICH. The hyperdense area of the affected vessel was correlated with CTA to establish the proximal portion of the occlusive hyperdense thrombus, when visible. Manual delineation was used to measure the length (in mm) and area (in mm^3^) of hyperdensity in the occluded vessel [16]. The infarct area was manually delineated on each CT slice (4 mm width), which yielded the area in cm^2^. The final infarct volume (FIV) in cm^3^ was calculated from the measured area and the corresponding slice thickness. Details of scanners, imaging protocols, and methodology for determining the area of hyperdense middle cerebral artery thrombus were reported previously [16]. Leptomeningeal collaterals were assessed on pre-procedural CT angiography and divided into three categories: 0—absence of collaterals in symptomatic hemisphere, 1—less visibility of collaterals in symptomatic hemisphere, 2—collaterals equal to non-symptomatic hemisphere [17].

A detailed timeline of intravenous thrombolysis (IVT) and endovascular treatment (EVT) was collected alongside the outcome of the recanalization attempt using the Thrombolysis In Cerebral Infarction (TICI) score [18]. Patients were divided into groups of unsuccessful or successful recanalization on the basis of TICI scores of 0, 1, and 2a; or 2b and 3, respectively.

We defined intracranial hemorrhage as per the Heidelberg Bleeding Classification [19]. We did not include patients with procedural ICH in our analysis, i.e., ICH clearly associated with the procedure itself, such as secondary to vessel perforation, arterial dissection, or subarachnoid hemorrhage.

Stroke etiology was established as per the Trial of Org 10172 in Acute Stroke (TOAST) criteria, following extensive workup [20].

Peripheral-venous blood was drawn routinely at the emergency room for assessment of complete blood count (CBC) and included leukocyte, neutrophil, and lymphocyte counts. As part of a standard of care procedure, additional lab tests were performed on the following day in fasting state to evaluate vascular risk factors. The neutrophil count divided by the lymphocyte count yielded the neutrophil to lymphocyte ratio (NLR). Additional variables included demographics, the National Institutes of Health Stroke Scale (NIHSS) score on admission and at discharge, and the modified Rankin Scale (mRS) at 3 months.

### Statistical analysis

Depending on the normality of distribution as assessed by the Kolmogorov-Smirnov test, continuous variables were compared using the *t* test for independent samples, or the Mann-Whitney *U* test. Categorical variables were compared using Fisher’s exact test or the Chi-square test. Binary logistic regression was performed to report odds ratios. All tests used a *p* value of 0.05 as a threshold for significance. The optimal cut-off value for the continuous NLR was calculated by applying a receiver operating curve analysis to test all possible cutoffs that would discriminate between ICH and no ICH. All statistical analyses were performed using STATA software 13.0 (StataCorp LLC, TX, USA).

## Results

Our initial cohort consisted of 204 patients. Eight patients were excluded because they had no follow-up imaging; three of these suffered early hospital death. Additional nine patients were excluded due to absence of laboratory data. Endovascular treatment was performed with stent-retriever devices except for three patients in whom aspiration technique was used.

The final analysis was therefore conducted for 187 patients. The median age of the cohort was 74 years (interquartile range (IQR) 60–81), and 86 (45.9%) were male. The pre-morbid mRS was > 2 in seven (3.7%) patients. The median NIHSS score at presentation was 18 (IQR 13–22). The time to first imaging after symptom onset was a median of 95 min (IQR 65–132). A total of 133 patients (71.1%) received intravenous thrombolysis. There were 37 (19.7%) deaths within the first 3 months from onset. Ninety (51.1%) patients had good outcome at 3 months, defined as a mRS of 0–2. The Alberta Stroke Program Early Computed Tomography Score (ASPECTS) at admission was < 8 in 32 patients (17.1%). The final infarct volume was determined in scans performed within 3 days (70%) and beyond 3 days (30%) from stroke onset. Procedure-related ICH caused by arterial perforation occurred in ten (5.1%) patients. We detected non-procedural ICH in 31 cases (16.6%); these patients also were less likely to experience a good outcome as compared to patients without a procedure-related ICH (*p* = 0.049). The ICH was symptomatic in 13 (8.6% of the entire cohort, and 41.9% of the non-procedural ICHs).

The median NLR was 3.6 (IQR 2.1–5.8). A higher NLR was not associated with the presence of malignancy (*p* = 0.662), chronic renal disease (*p* = 0.814), or heart failure (*p* = 0.868). As compared to patients without an ICH, those having sustained an ICH had higher NLR levels (*p* = 0.003, 5.1 vs. 3.2) and a worse outcome at 3 months as measured with the mRS (*p* = 0.013, 5.3 vs. 4.0). Blood glucose level on admission, leptomeningeal collateral status, and the number of passes during thrombectomy were not different between the groups with or without an ICH. We found, however, that more than three passes of the catheter was associated with sICH (20.4% (> 3 passes) vs. 4.3% (≤ 3 passes); *p* = 0.002). Further characteristics of patients with and without ICH are presented in Table 1.

**Table 1:** Characteristics of 195 patients who underwent EVT for recanalization of large artery anterior circulation ischemic stroke

	All (*N* = 187)	ICH (*N* = 31)	No ICH (*N* = 156)	*p*
Age, median (IQR)	74 (60–81)	77 (52–84)	73 (62–80)	0.592
Male sex	86 (45.9)	12 (38.7)	19 (61.3)	0.433
Hypertension	119 (63.6)	20 (64.5)	99 (63.5)	1.000
Diabetes mellitus	24 (12.9)	3 (9.7)	21 (13.5)	0.771
Atrial fibrillation	112 (59.9)	15 (48.4)	16 (51.6)	0.165
Cancer	8 (4.4)	1 (3.2)	7 (4.6)	1.000
Chronic kidney failure	11 (6.1)	1 (9.1)	10 (6.6)	0.694
Chronic heart failure	25 (13.9)	6 (24)	20 (12.8)	0.371
Admission values				
Pre-morbid mRS > 1	7 (3.7)	3 (9.7)	4 (2.6)	0.091
Body mass index (*N* = 156)	25.4 (23.1–29.0)	24.2 (21.9–27.8)	25.7 (23.4–29.0)	0.058
NIHSS	18 (13–22)	18 (14–23)	18 (13–22)	0.311
Occlusion site				0.064
M1	145 (77.5)	20 (64.5)	125 (80.1)	
ACI + M1/M2	22 (22.5)	11 (35.5)	31 (19.9)	
ASPECTS	8 (8–9)	8 (7–9)	9 (8–9)	0.226
Serum glucose	118 (107–142)	123 (112–150)	118 (106–142)	0.290
Neutrophil to lymphocyte ratio	3.6 (2.1–5.8)	5.1 (2.9.-8.4)	3.2 (1.9–5.2)	*0*.*003*
Hyperdense thrombus area (*N* = 105)	25.7 (15.3–45.2)	34.0 (25.1–52.7)	22.3 (13.6–41.8)	*0*.*039*
Stroke etiology				0.075
Cardioembolic and unknown	153 (81.8)	29 (93.5)	124 (75.4)	
Large artery atherosclerotic + other etiology	34 (18.2)	2 (6.4)	32 (20.5)	
Procedure related				
Intravenous thrombolysis	133 (71.5)	21 (67.7)	112 (72.3)	0.664
Time to first imaging, min	95 (65–132)	107 (83–155)	88.5 (64–128)	*0.045*
Time to needle	112 (85–150)	135 (110–167)	105 (82–140)	*0*.*034*
Time to groin puncture	189 (148–233)	205 (180–252)	186 (144–227)	0.073
Number of passes > 3	76 (50.0)	16 (59.3)	60 (48.0)	0.396
Intervention time	52 (25–93)	70 (35–116)	50 (21–85)	0.059
ICA Stenting	15 (28.3)	2 (22.2)	13 (29.5)	1.000
TICI 2b or 3	141 (75.8)	20 (64.5)	11 (35.5)	0.114

Numbers are medians with interquartile range in parentheses or number (percentage)

*EVT* endovascular treatment, *NIHSS* National Institutes of Health Stroke scale, *mRS* modified Rankin scale, *ASPECTS* Alberta Stroke Program Early Computed Tomography Score, *ICA* internal carotid artery, *MCA* middle cerebral artery, *TICI* thrombolysis in cerebral infarction

Patients with a pre-morbid mRS of > 1 were not more frequent in the group that developed ICH (*p* = 0.091). There was a trend for a lower body mass index (BMI) in patients with ICH; however, it did not reach statistical significance (median 24.2 vs. 25.7; *p* = 0.058). The size of the thrombus measured as the area in cm^2^ of the hyperdense portion of MCA was significantly larger in patients with ICH (*N* = 105, median 5.06 vs. 3.23 cm^2^; *p* = 0.039). Tandem occlusion of the ICA and MCA (ICA cervical occlusion and ICA distal to bifurcation together with MCA occlusion) showed a trend toward an increased risk of ICH (35.5% vs 19.9%; *p* = 0.064). We found a significantly higher NLR in patients with ICH (median 5.1 vs. 3.2; *p* = 0.003). A longer time to thrombolysis and time to first imaging were more common in patients with ICH (median 135 vs. 105 and median 107 vs. 88 with *p* = 0.034 and *p* = 0.045, respectively). Absence of sufficient reperfusion (TICI score 0-2a) was not significantly different between the two groups (64.5 vs. 35.5%; *p* = 0.114).

In multivariate logistic regression analysis adjusted for age, site of vessel occlusion (ICA ± MCA vs. MCA alone), and intervention time in minutes, NLR was independently associated with ICH (OR 1.09 per unit increase, 95% CI 1.00–1.20; *p* = 0.040). Further details are shown in Table 2.

**Table 2:** Results of multivariate logistic regression analysis of predictors for intracranial hemorrhage in 187 patients with endovascular treatment due to large artery occlusion of the anterior circulation

Variables	Values	Odds ratio (95% confidence interval)	*p*
Age	Years	0.99 (0.96–1.02)	0.628
Occlusion site	M1 vs. ICA ± M1/2	1.73 (0.70–4.27)	0.232
Intervention time	By 1 min increase	1.00 (0.99–1.01)	0.192
Neutrophil/lymphocyte ratio	By 1 unit increase	1.09 (1.00–1.20)	*0*.*040*

*M1* M1 segment of middle cerebral artery, *ICA* internal carotid artery

**Fig. 1:**
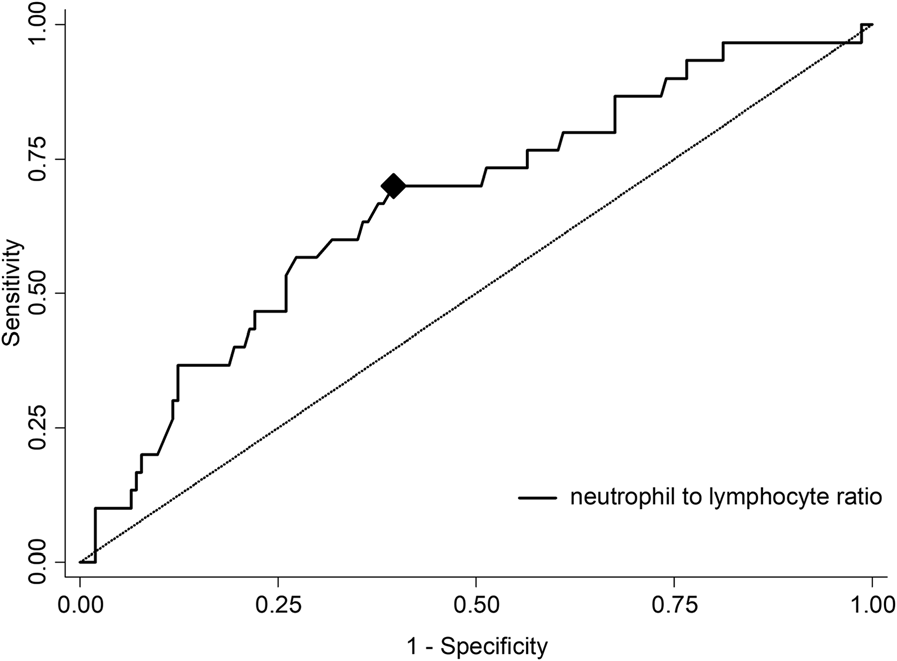
Discriminative ability presented as receiver operating curve of neutrophil to lymphocyte ratio (NLR) to predict intracranial hemorrhage. Area under curve: 0.67. Diamond representing cut-off NLR value of 3.89

The area under the curve (AUC) for the ability of the NLR at admission to predict ICH was 0.67 with 67.0% sensitivity and 73.0% specificity (Fig. 1). The optimal cutoff value of NLR that best distinguished the development of ICH was 3.89.

## Discussion

In this single-center retrospective study of acute ischemic stroke caused by large vessel occlusion and treated with EVT, we detected non-procedural ICH and sICH rates of 16.6% and 8.6%, respectively. In comparison to large EVT trials, our ICH rates are higher, probably reflecting real-world setting of our study [3]. Most importantly, we could translate previous findings in acute ischemic stroke regarding the predictive value of NLR, a systemic subclinical marker of inflammation, to the development of ICH in EVT-treated ischemic stroke caused by large vessel occlusion. In this regard, we corroborate and expand recent findings by Goyal et al. in that higher admission NLR is an independent predictor of sICH and 3-month mortality [21]. NLR measured at admission in a previous cohort of 143 patients with ischemic stroke and a median NIHSS of 6 predicted 3-month outcome, with a cut-off value for poor outcome of 2.99 [13]. Our study suggested a cut-off for ICH of 3.89 (67.0% sensitivity and 73.0% specificity), whereas the aforementioned study calculated best predictive cut-off values of admission NLR for sICH and 3-month mortality of 6.62 (sensitivity 71% and specificity 76%) and 4.29 (sensitivity 59% and specificity 56%), respectively. We found a median NLR of 3.6 in our cohort, which is much higher than the NLR ratios found in the healthy population. For instance, a large study in healthy Koreans with a median age of 47 years reported a mean NLR ratio of 1.65 [22]. Another study of an adult healthy population reported a median NLR of 1.65 and a range of 0.78–3.53 [23]. This rise of the NLR in acute ischemic stroke is believed to result from an activation of neutrophils (set out to infiltrate brain parenchyma) and a suppression of lymphocytes by systemic stress [24]. The associated increased release of MMP-9, disruptive for the neurovascular unit, is one of the pathophysiological explanations for the development of ICH and poor outcome in patients with high NLR. The role of neutrophils becomes even more complex as pro-inflammatory N1 neutrophils are implicated in brain edema and neurotoxicity, whereas anti-inflammatory N2 neutrophils were found to limit this excessive immune response promoting neuronal survival and successful brain remodeling [25]. There is a paucity of studies, however, investigating factors associated with hemorrhagic transformation in general, and particularly in the setting of EVT. A recent study performed in an Asian population of 632 patients treated with EVT for anterior circulation stroke showed a high occurrence of sICH of 16% [7]. The occurrence of sICH was independently associated with a baseline NLR ratio of > 0.83, pretreatment ASPECTS of < 6, delayed recanalization treatment, multiple device passes, stroke of cardioembolic type, and poor collateral circulation. These findings are likely to be influenced by the significant number of intra-arterial use of rt-PA or tirofiban in that study. In addition, the reported NLR is well under normal values, therefore its interpretation should warrant some caution. Maestrini et al. studied baseline NLR in 846 patients (median NIHSS 10) treated with rt-PA, and found an independent association with sICH (6.4%), death, and worse outcome at 3 months [15]. They calculated a NLR ≥ 4.80 (sensitivity 66.7%, specificity 71.3%) as the discriminative threshold.

We could not confirm systemic rt-PA treatment as a risk factor for ICH in our EVT-treated cohort. We had a rt-PA rate of 71%, and a door to needle time of a median of 112 min (IQR 85–150). A study by Guo et al. analyzed 189 patients with acute ischemic stroke, a third of whom were also treated with EVT [26]. Interestingly, NLR measured at admission was not predictive of ICH or sICH. The main finding of that, however, was an independent association of NLR obtained at 12–18 h post-treatment with ICH and sICH, with NLR > 10.59 leading to a 8.50-fold greater risk of ICH, and a 7.93-fold increased risk for sICH, although the confidence intervals were wide (2.69–26.89 and 2.25–27.99, respectively). In our sample, NLR at admission had lower sensitivity and specificity for predicting ICH (67% and 73%, respectively), and the cut-off value was significantly lower. Furthermore, only 13 (7%) of our patients were above the proposed cut-of value of Guo et al. The differences between the two studies include the time of NLR determination and treatment approaches, with all our patients receiving EVT that is superior to rt-PA alone. Xue et al. [11] and Fang et al. [27] reported a correlation between NIHSS and NLR determined at admission in 280 and 1731 stroke patients, respectively, but the treatment modalities were not reported. We could not confirm these findings since NLR was not correlated with stroke severity at admission in our cohort. The discrepancy most probably is due to the fact that Xue et al. recruited patients experiencing stroke within 6 days before admission, and Fang et al. within 48 prior to admission. In addition, Xue et al. found association of NLR with primary unfavorable outcome, similar to our cohort, and increased risk for recurrent stroke with a hazard ratio of 1.499 (95% CI 1.161–1.935, *p* = 0.002). Fang et al. observed that patients with an NLR ≥ 3.2 had a 2.55-fold risk of in-hospital mortality. Neither of these studies however reported on the incidence of ICH or sICH.

Reports describing an association of BMI with stroke outcome have been controversial. In the setting of rt-PA treatment, BMI had no prognostic significance for sICH or the 3-month outcome [28]. Another study suggested underweight state as risk factor for unfavorable outcome [29].Using standard groups of BMI categories (underweight < 18.5 kg/m^2^, reference 18.5–24.9 kg/m^2^, overweight 25.0–29.9 kg/m^2^, and obese > 30.0 kg/m^2^), our patients in the underweight group (*N* = 9) had a 33.3% chance for ICH, whereas this risk was the smallest for the overweight group at only 5% (*N* = 59). The association of BMI with ICH is most likely multifactorial, and not necessarily causative. Tandem occlusions are known predictors for sICH with up to 22.0% of patients affected after EVT [30]. Larger clot burden could impair penumbral perfusion and damage the vessel wall itself, leading to vessel disruption and intracerebral hematoma [31]. Leptomeningeal collaterals were shown to protect against ICH, but in our study the absence of collaterals was not associated with ICH or sICH [32]. The reason for this is not clear since the collateral status had a significant influence on final infarct volume and 3-month outcome. We had a rate of 5.1% for procedure-related ICH, which is in a similar range reported in the REVASCAT trial (4.9%). In four other trials, however, the rate of arterial perforation ranged from 0 to 2.9% [33].

There are some limitations to our study. There is retrospective bias inherent to the study design, and also there were prolonged times to imaging and rt-PA in patients who developed ICH. Future studies should also take immunologically relevant comorbidities such as sarcopenia and rheumatoid arthritis into account.

## Conclusions

We found NLR at admission to be an independent predictor of non-procedural ICH in acute ischemic stroke caused by large vessel occlusion when treated with EVT. Our findings in a moderate-sized retrospective cohort highlight the need to better understand post-stroke immune response and confirm the value of NLR as a potential biomarker of a subgroup at risk for ICH.

## Abbreviations

ASPECTS: Alberta stroke programme early CT score
BMI: Body mass index
CT: Computed tomography
EVT: Endovascular therapy
ICH: Intracerebral hemorrhage
NIHSS: National Institute of Health Stroke Scale
NLR: Neutrophil to lymphocyte ratio
rt-PA: Recombinant tissue-plasminogen activator
